# Role of CTCF Protein in Regulating *FMR1* Locus Transcription

**DOI:** 10.1371/journal.pgen.1003601

**Published:** 2013-07-18

**Authors:** Stella Lanni, Martina Goracci, Loredana Borrelli, Giorgia Mancano, Pietro Chiurazzi, Umberto Moscato, Fabrizio Ferrè, Manuela Helmer-Citterich, Elisabetta Tabolacci, Giovanni Neri

**Affiliations:** 1 Istituto di Genetica Medica, Università Cattolica del S. Cuore, Rome, Italy; 2 Istituto di Igiene, Università Cattolica del S. Cuore, Rome, Italy; 3 Dipartimento di Biologia, Università di Roma “Tor Vergata”, Rome, Italy; The Hospital for Sick Children and University of Toronto, Canada

## Abstract

Fragile X syndrome (FXS), the leading cause of inherited intellectual disability, is caused by epigenetic silencing of the *FMR1* gene, through expansion and methylation of a CGG triplet repeat (methylated full mutation). An antisense transcript (*FMR1*-*AS1*), starting from both promoter and intron 2 of the *FMR1* gene, was demonstrated in transcriptionally active alleles, but not in silent FXS alleles. Moreover, a DNA methylation boundary, which is lost in FXS, was recently identified upstream of the *FMR1* gene. Several nuclear proteins bind to this region, like the insulator protein CTCF. Here we demonstrate for the first time that rare unmethylated full mutation (UFM) alleles present the same boundary described in wild type (WT) alleles and that CTCF binds to this region, as well as to the *FMR1* gene promoter, exon 1 and intron 2 binding sites. Contrariwise, DNA methylation prevents CTCF binding to FXS alleles. Drug-induced CpGs demethylation does not restore this binding. *CTCF* knock-down experiments clearly established that CTCF does not act as insulator at the active *FMR1* locus, despite the presence of a CGG expansion. *CTCF* depletion induces heterochromatinic histone configuration of the *FMR1* locus and results in reduction of *FMR1* transcription, which however is not accompanied by spreading of DNA methylation towards the *FMR1* promoter. *CTCF* depletion is also associated with *FMR1-AS1* mRNA reduction. Antisense RNA, like sense transcript, is upregulated in UFM and absent in FXS cells and its splicing is correlated to that of the *FMR1*-mRNA. We conclude that CTCF has a complex role in regulating *FMR1* expression, probably through the organization of chromatin loops between sense/antisense transcriptional regulatory regions, as suggested by bioinformatics analysis.

## Author Summary

Fragile X syndrome is the most common cause of inherited intellectual disability, accounting for about 1∶3000 males and 1∶4000 females. It is caused by a dynamic mutation of *FMR1*, a gene mapping on the X chromosome and containing a CGG repeat in its promoter region. Expansion of this unstable sequence beyond 200 repeats (full mutation) is followed by DNA methylation and histone changes, leading to the transcriptional inactivation of *FMR1* and to the lack of the FMRP protein. Recently, an antisense transcript (*FMR1-AS1*) spanning the CGG repeats and a region of transition of DNA methylation (boundary) located upstream of the CGG repeats have been identified in transcriptional active *FMR1* alleles. Several nuclear proteins bound to the methylation boundary have been described, such as the zinc-finger protein CTCF, the first known insulator in mammals. This protein is an important transcriptional regulator of genes harboring trinucleotide repeats and it is mostly active in chromatin organization. For the first time, we have investigated the role of CTCF protein in the transcriptional regulation of the *FMR1* gene. Our results define a complex role for CTCF acting through chromatin organization of the *FMR1* locus.

## Introduction

Fragile X syndrome (FXS, OMIM #300624), the most studied and best known FRAXopathy, is the leading cause of inherited intellectual disability (ID) [1]. FXS is caused by the expansion beyond 200 repeats (full mutation) and subsequent methylation of the polymorphic CGG sequence within the 5′ untranslated region (5′ UTR) of the *FMR1* gene, an X-linked gene which contains a CpG island in its promoter [2]. The methylation of cytosines of both the expanded CGGs and of the neighboring CpGs, as well as other heterochromatic histone modifications, cause the transcriptional silencing of the *FMR1* gene and the lack of the FMRP protein [3], [4]. FMRP is an RNA-binding protein, which inhibits the translation of messenger RNAs (mRNAs), especially within post-synaptic vesicles of the dendritic spines. Its absence impairs synaptic plasticity, which is thought to be the cause of ID [5]. Previous reports described rare individuals of normal intelligence, carrying a transcriptionally active unmethylated full mutation (UFM) [6]–[8]. Cell lines derived from these individuals might reflect the status of FXS cells before epigenetic silencing, that is thought to occur at about 11 weeks of gestation [9]. Indeed, the epigenetic characterization of their *FMR1* locus showed histone H3 and H4 hyperacetylation, lysine 4 of histone 3 (H3-K4) methylation, lysine 9 of histone 3 (H3-K9) hypomethylation, lysine 27 of histone 3 (H3-K27) dimethylation and lack of DNA methylation [7], [8]. This epigenetic status is compatible with an euchromatic conformation of the *FMR1* locus, allowing transcription. A similar epigenetic status can be induced by treatment of FXS cells with the DNA demethylating agent 5-aza-2-deoxycytidine (5-azadC), which also causes histone changes (hyperacetylation, H3-K4 methylation), the latter actually preceding DNA demethylation [4], [10], [11]. In accordance with these results, silencing of *FMR1* in human embryonic stem cells seems to begin from histone modifications prior to DNA methylation [12].

In FXS cell lines DNA methylation extends further to approximately 1 kb upstream the CGG repeat sequence [13]. In wild-type (WT) alleles a zone of transition between methylated and unmethylated sequences was described around 650 to 800 nucleotides upstream the CGG repeat, with CpGs being unmethylated all the way down to the *FMR1* promoter. This methylation boundary (MB) appears to be lost in completely methylated FXS alleles. The boundary is also conserved in the mouse genome, even if human and mouse are only 46.7% identical in the 5′ region upstream the *FMR1* gene [13].

Methylation boundary regions are characterized by the presence of binding sites for various nuclear proteins including CTCF (CCCTC-binding factor), the first insulator protein found in mammals [14]. CTCF is a widely expressed nuclear protein, which binds different DNA target sequences through its 11 zinc-finger domains [15], [16]. It was first discovered as a negative transcriptional regulator, interacting with various sequences in the promoter of the chicken, mouse and human *C-MYC* oncogene [17], [18]. Subsequent studies recognized its involvement in several functions, including transcriptional activation or repression, X chromosome inactivation, genomic imprinting, methylation-dependent chromatin insulation and higher-order chromatin organization through the establishment of DNA loops [19]–[22]. CTCF has been implicated in the organization of both the structure of the chromosomal fiber within each individual chromosome and of the chromosome territories within the cell nucleus. Many CTCF binding sites reside within promoters, as well as in inter- and intra-genic regions [23]. The relationship between CTCF binding patterns and DNA methylation is currently unknown. Pre-existing methylation can antagonize CTCF binding *in vitro* [24]–[26]. A recent study of overall methylation status showed that 98% of CTCF sites were unmethylated in at least one of the 13 cell types tested, confirming an inverse relationship between DNA methylation and CTCF occupancy [27]. Despite that, it is still unclear whether demethylation facilitates subsequent CTCF binding and whether bound CTCF maintains the corresponding domain in an unmethylated status.

An important regulatory role of CTCF was described in expanded triplet diseases. Specific binding sites for this protein were recognized flanking the CTG triplet at the *DM1* locus of myotonic dystrophy [28]. Recent evidence suggests that both CTCF binding and CpG methylation may contribute to CTG repeats instability [29], [30]. In a transgenic mouse model for spinocerebellar ataxia type 7 (SCA7), CTCF regulates ataxin-7 gene expression and is required for *SCAANT1* (SCA7 antisense noncoding transcript 1) expression. Loss of *SCAANT1* de-represses ataxin-7 sense transcription in a cis-dependent manner and is accompanied by chromatin remodeling [31]. In Friedreich ataxia (FRDA), caused by expansion of a GAA repeat sequence in intron 1 of the *FXN* gene, CTCF depletion was observed in the 5′ UTR of the mutant alleles. This depletion is associated with high levels of the transcript antisense of *FXN* (*FAST-1*), supporting the hypothesis of an epigenetic silencing of the corresponding “sense” gene [32].

Four CTCF binding sites have been identified within the *FMR1* locus, suggesting a role of this protein in the regulation of the gene [33]. In the same report, an antisense transcript of the *FMR1* gene (*FMR1-AS1*) spanning the expanded CGG repeat was identified in normal and premutated alleles, but not in FXS alleles. The authors suggested a possible pathogenic role of *FMR1-AS1* in FXS and also in the fragile X tremor-ataxia syndrome (FXTAS) associated with premutated alleles. However, they did not study the presence of the antisense transcript in UFM cells.

In this paper we investigate the role of CTCF in transcriptional regulation of the *FMR1* gene and in chromatin organization of the corresponding locus including the methylation boundary region, in different cell lines derived from normal (WT), FXS and UFM individuals, respectively. Through molecular and bioinformatics approaches we demonstrate that CTCF does not preserve the methylation boundary of the *FMR1* locus, but is required for its proper transcription. Significant results were obtained from the further characterization of the rare UFM cell lines by mapping the methylation boundary region and by measuring the *FMR1* antisense transcript.

## Results

### Identification of methylation boundary and *FMR1*-*AS1* in UFM cell lines

The extended region upstream the CGG repeats described by Naumann et al. [2009] [13] was analyzed in three classes of cell lines (WT, FXS and UFM), both lymphoblasts and fibroblasts. Bisulfite sequencing of the methylation boundary in WT cell lines confirmed the results already reported [13], with a DNA methylation boundary located at CpG pairs 70–71 in lymphoblastoid cells (**Figure S1A**) and 73–74 in fibroblasts (**Figure S1B**), respectively. As expected, no boundary was present in FXS cells. Despite the presence of the CGG expansion, the transcriptionally active UFM cell lines retained the methylation boundary as in WT cells, both in lymphoblasts and in fibroblasts (**Figure S1A** and **B**).

**Figure 1:**
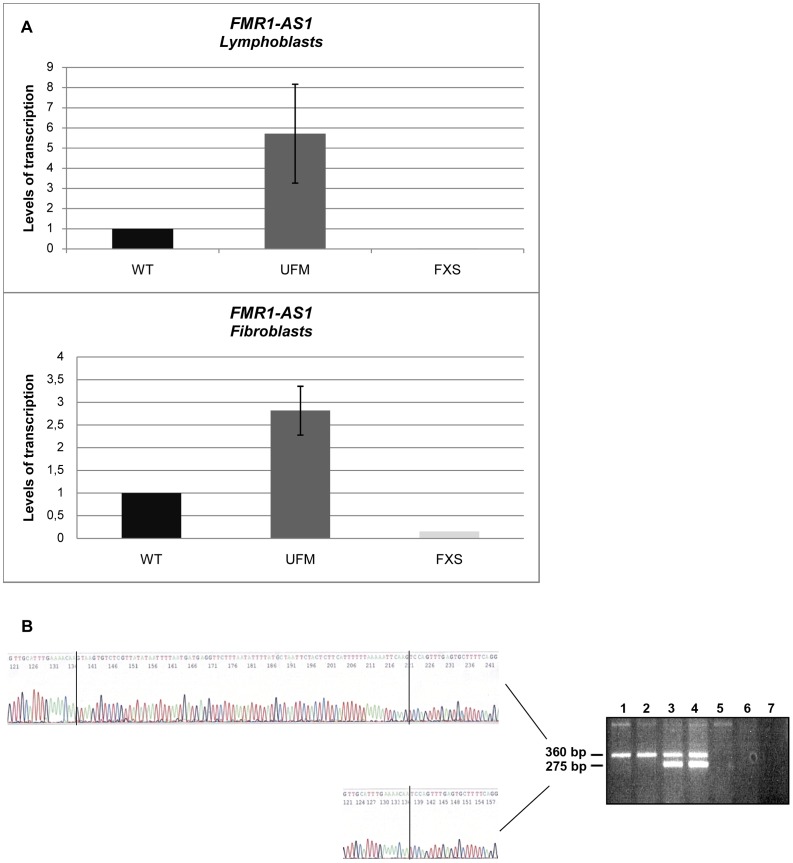
Characterization of *FMR1-AS1* transcript in UFM cell lines. (**A**) Quantification of antisense transcript through RT-PCR in WT, UFM and FXS lymphoblasts (n = 5) and fibroblasts (n = 4). (**B**) Strand specific RT-PCR (on the right) spanning the position +10243 to +210 bp (Genbank L29074) in lymphoblastoid cells: WT (lanes 1 and 2), UFM (lanes 3 and 4), FXS (lanes 5 and 6), no template control (lane 7). Sequencing analysis (on the left) representing the two isoforms of antisense transcript identified in UFM cells with the non-consensus CT to AC splice site in the intron 1 (360 bp isoform) and 2 (275 bp isoform) of *FMR1* gene.

We went on to quantify *FMR1-AS1* transcript levels and observed higher levels of transcription in UFM lymphoblasts (around 6-fold higher) and fibroblasts (around 3-fold higher) compared to WT, while no antisense transcript was detected in FXS cells, as expected [33] (**Figure 1A**). These results showed that the antisense transcript follows the same expression pattern as the sense RNA [8]. Amplification and sequencing analysis of *FMR1-AS1* cDNA in WT and UFM cells confirmed the presence of the splicing corresponding to the intron 1 of the sense transcript (**Figure 1B**), despite the recognition of a non-canonical AC-CT splice site in the antisense mRNA. Moreover, UFM cells presented a second isoform of antisense transcript, which retained the non-canonical splicing in intron 2, like in premutation alleles [33] (**Figure 1B**). Based on *FMR1-AS1* data, we may hypothesize a co-regulation mechanism for sense and antisense transcription at the *FMR1* locus.

### CTCF binding to *FMR1* locus is not restored after DNA demethylation

**Figure 2:**
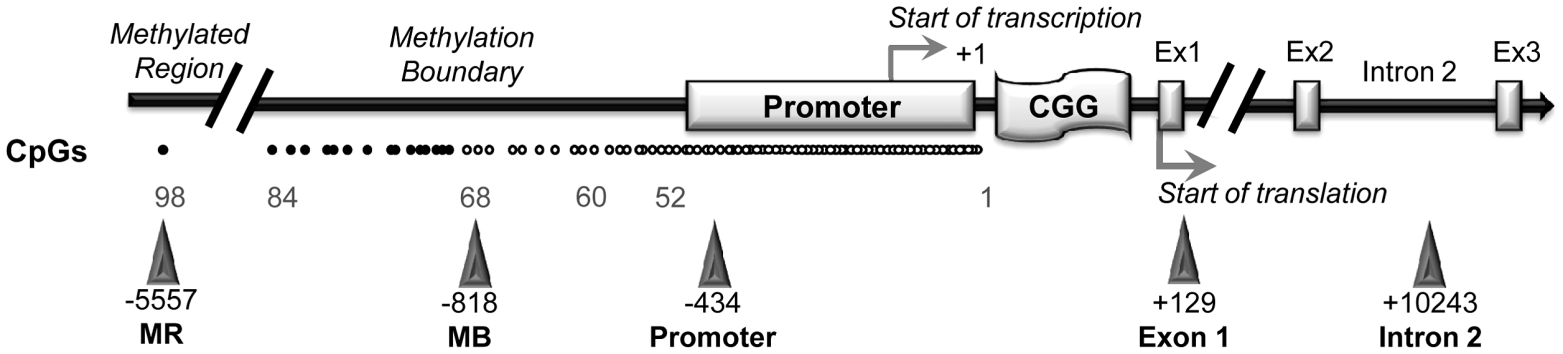
CTCF binding sites on *FMR1* locus. A schematic outline of CTCF binding sites spanning the *FMR1* locus (white dot = unmethylated CpG; black dot = methylated CpG). Triangles indicate CTCF binding sites. Promoter, exon 1, intron 2 and methylation boundary (indicated as MB) sites had been previously described [33]; the site present in the upstream methylated region (indicated as MR) was identified through the database available online [34]. The transcription start site is reported as +1, as referred to Genbank L29074.

**Figure 3:**
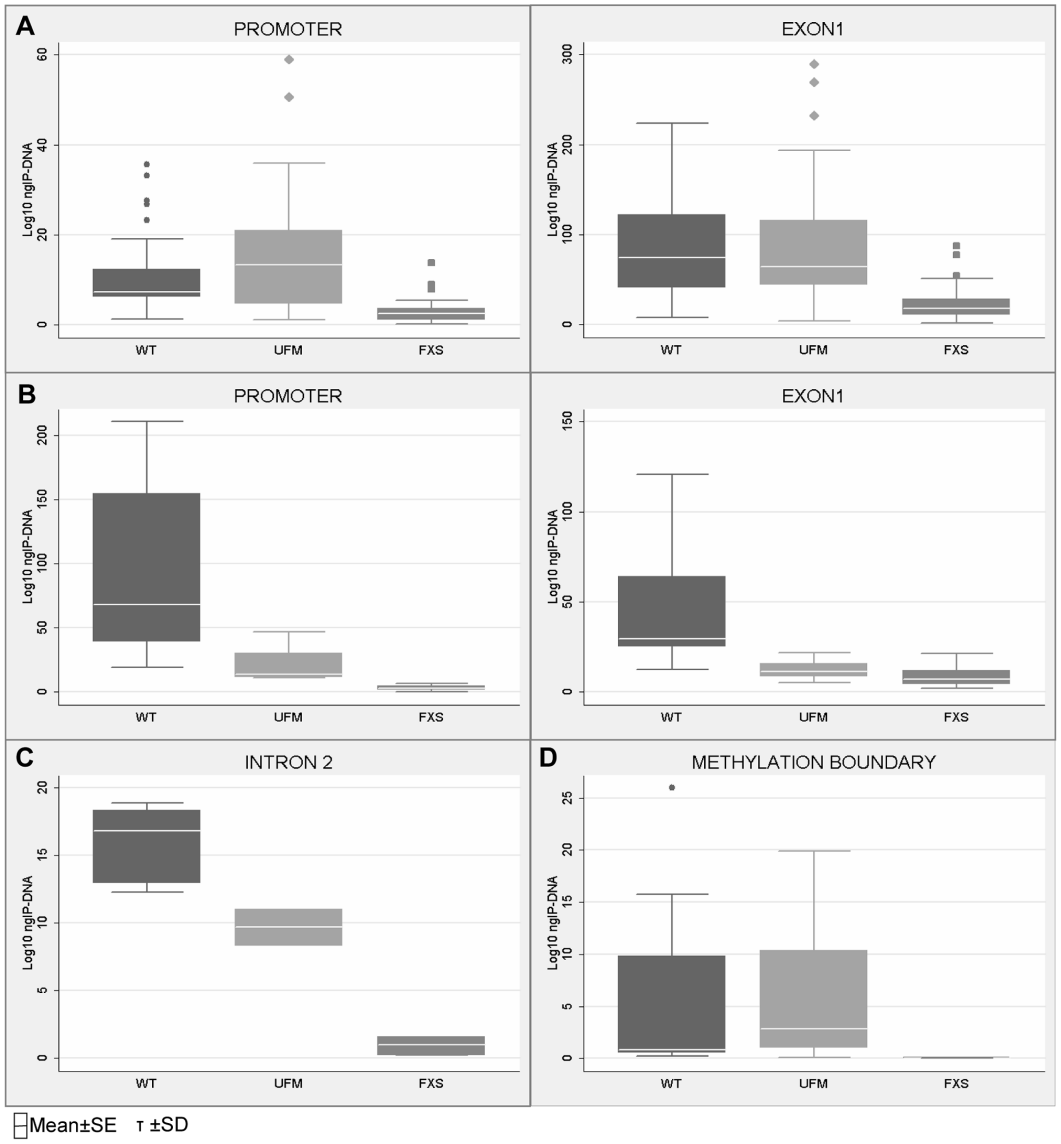
Quantification of CTCF binding on *FMR1* locus through ChIP analysis. ChIP assay of CTCF binding on *FMR1* promoter and exon 1 in WT, UFM and FXS cell lines, both lymphoblasts (**A**) and fibroblasts (**B**). Box-plots indicate the mean of at least 10 independent immunoprecipitations for each lymphoblastoid cell line and 3 for fibroblasts. For each region analyzed the levels of CTCF binding in WT and UFM lines was significantly higher compared to FXS lines (p = 0.0001 for lymphoblasts and p<0.05 for fibroblasts). ChIP assay of CTCF binding on intron 2 (**C**) and methylation boundary region (MB) (**D**) in WT, UFM and FXS fibroblasts. The level of CTCF binding in WT and UFM lines is significantly higher compared to FXS lines (p = 0.0001) for both regions analyzed. Box-plots indicate the mean of three independent experiments for the MB binding site and one for the intron 2 site. Note that the amount of IP-DNA (ng) is indicated in logarithmic scale. In all ChIP experiments negative controls were performed by IgG immunoprecipitation and no template control (not shown).

CTCF binding sites on the *FMR1* gene were previously reported [33]. We now include one additional site obtained from the database available online at http://insulatordb.uthsc.edu/ [34], designated MR (methylated region) site, located at −5557 bp upstream the *FMR1* transcription start site. A schematic outline of all CTCF binding sites within the *FMR1* locus included in our study is represented in **Figure 2**. We first studied the three CTCF binding sites in the promoter and near exon1, flanking the CGG repeat sequence, and in the intron 2 region, near one of the transcription starting site of *FMR1-AS1* in UFM cell lines. ChIP assay results demonstrated the binding of CTCF to these three sites in UFM fibroblasts and lymphoblasts (**Figure 3A–C**). The level of binding in UFM was significantly higher compared to FXS cells, both fibroblasts and lymphoblasts, in all sites analyzed. In promoter and exon 1 regions lymphoblasts showed similar CTCF binding levels in UFM and WT (**Figure 3A**), while in WT fibroblasts CTCF binding levels were significantly higher (p<0.05) compared to UFM (**Figure 3B**).

In WT cells, we confirmed CTCF binding to the MB site between CpG pairs 66–69. As expected, no CTCF binding was found in FXS fibroblasts, given the complete methylation of this region. Instead, UFM fibroblasts showed binding levels similar to those of WT cells (**Figure 3D**), demonstrating that CTCF binding is strictly related with the unmethylated status of *FMR1* locus. The MR binding site at −5557 bp corresponds to CpG 98, which is fully methylated in all cell lines under investigation. Expectedly, we did not detect CTCF binding in any of them, both fibroblasts and lymphoblasts (data not shown).

**Figure 4:**
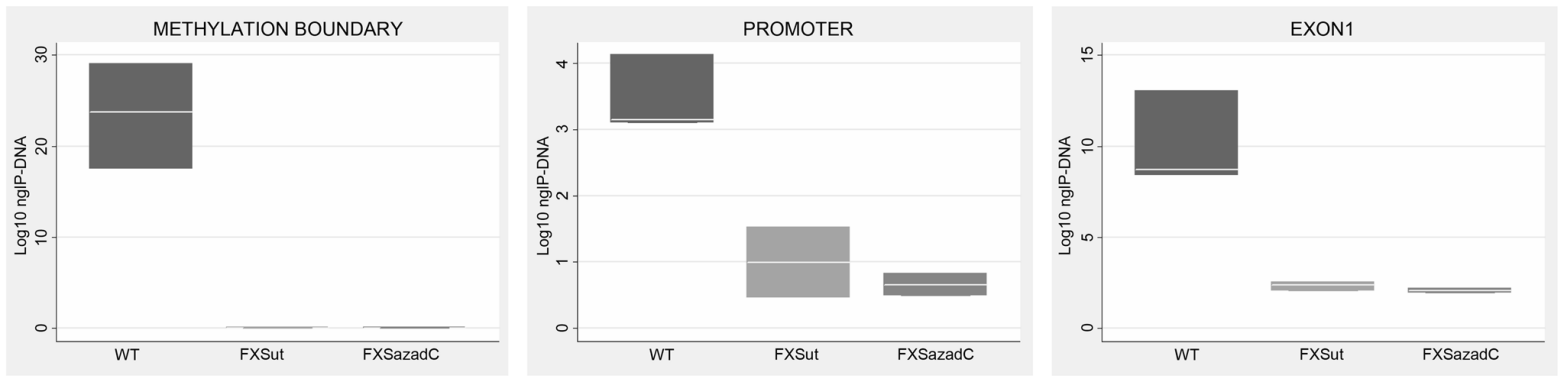
CTCF binding on *FMR1* locus through ChIP analysis after pharmacological demethylation. ChIP assay of CTCF binding to *FMR1* methylation boundary, promoter and exon 1 region after a 7-day treatment with [1 µM] 5-azadC on the S1 (FXS) lymphoblastoid cell line with 450 CGGs. After the pharmacological treatment we observed 25% of *FMR1* transcriptional reactivation. Box plots represent the amount of DNA bound by CTCF in untreated WT lymphoblasts, untreated FXS (FXSut) and FXS treated with 5-azadC (FXSazadC) cell lines. Note that the amount of IP-DNA (ng) is indicated in logarithmic scale. ChIP experiments included negative controls performed by IgG immunoprecipitation and no template control (not shown).

We speculated that after DNA demethylation CTCF might rebind to its sites on the *FMR1* locus in FXS cells. Our previous studies demonstrated that treatment of FXS lymphoblastoid cells with the demethylating agent 5-azadC induces *FMR1* transcriptional reactivation, consequent to demethylation of the 52 CpGs of the promoter [10], [11]. After a 7 day-treatment with 5-azadC of a FXS lymphoblastoid line, we did not observe any significant change in cell viability. We obtained a 25% transcriptional reactivation of *FMR1* and a related eight-fold increase of *FMR1-AS1* transcript (data not shown). However, as indicated in **Figure 4**, 5-azadC treatment did not restore CTCF binding to the reactivated *FMR1* gene in exon 1, promoter and boundary region (MB site).

### CTCF involvement in *FMR1* transcriptional regulation

After demonstrating that CTCF binds to the *FMR1* regulatory region in transcriptionally active cells, we went on to investigate whether CTCF protein had a regulatory function in *FMR1* gene transcription.

**Figure 5:**
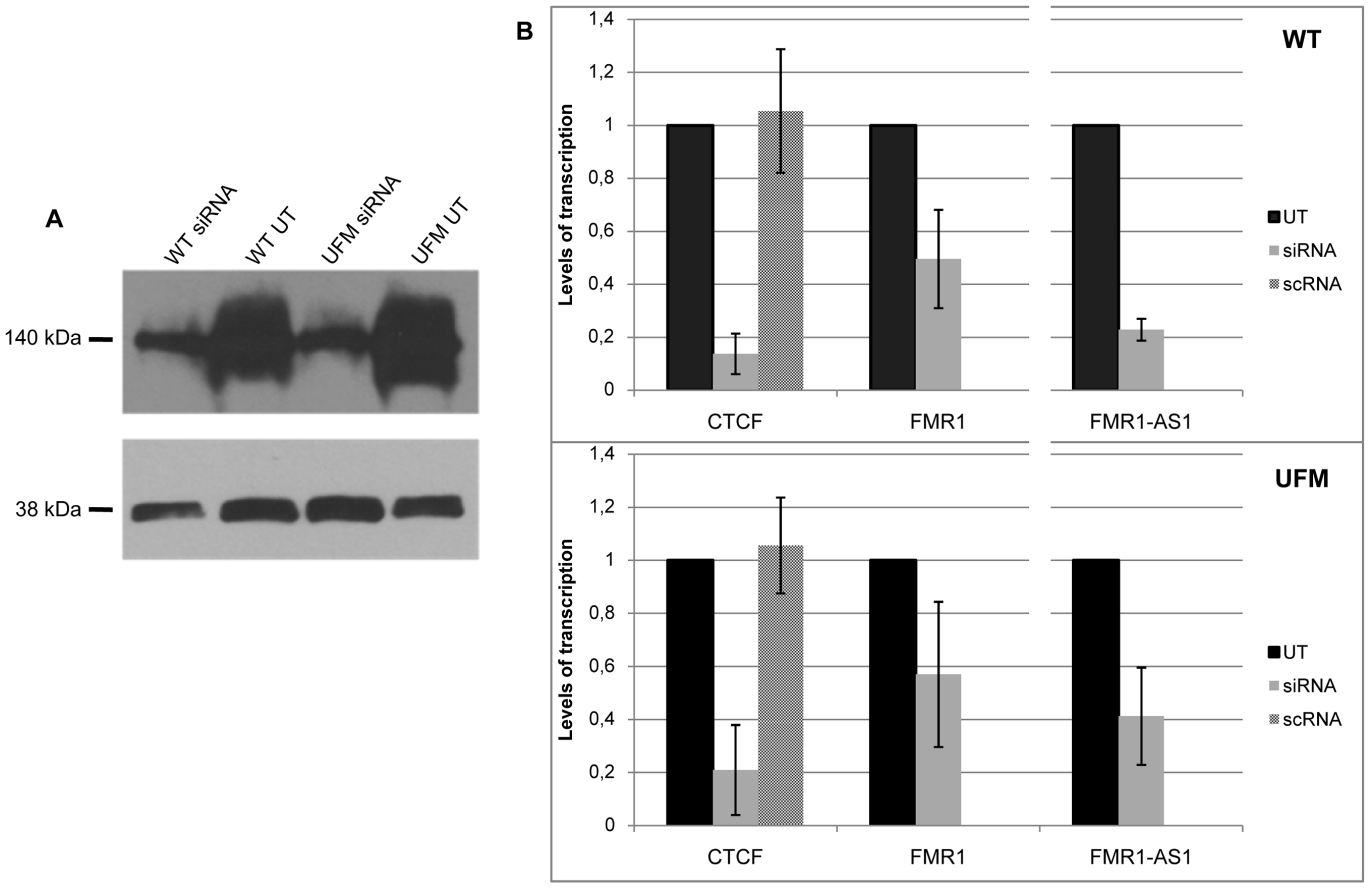
*CTCF* knock-down in WT and UFM fibroblasts. (**A**) Western blotting analysis of CTCF, visualized with ECL kit. Protein extracts from untreated (UT) and siRNA-treated WT and UFM fibroblasts were probed with an anti-CTCF rabbit polyclonal antibody (top panel) and one against GAPDH (bottom panel). After *CTCF* depletion a major reduction of the corresponding protein is visible. (**B**) Relative quantification through RT-PCR of *CTCF* and *FMR1* sense and antisense transcripts in those knock-down experiments in which *CTCF* depletion is followed by *FMR1* transcript reduction both in WT (upper panel) and UFM (bottom panel) fibroblasts. *FMR1* sense transcript is reduced to around 50% in both cell lines with a consistent (about 80%) reduction of *CTCF* mRNA levels. Depletion of *FMR1*-*AS1* (80 and 60% in WT and UFM cells, respectively) is also observed and directly correlates with *CTCF* reduction in both cell lines. The two cell lines were also transfected with a scramble siRNA (scRNA) without any modifications of *CTCF* transcript levels. Histograms represent mean and standard deviation of 10 independent knock-down experiments for UFM and WT.

**Figure 6:**
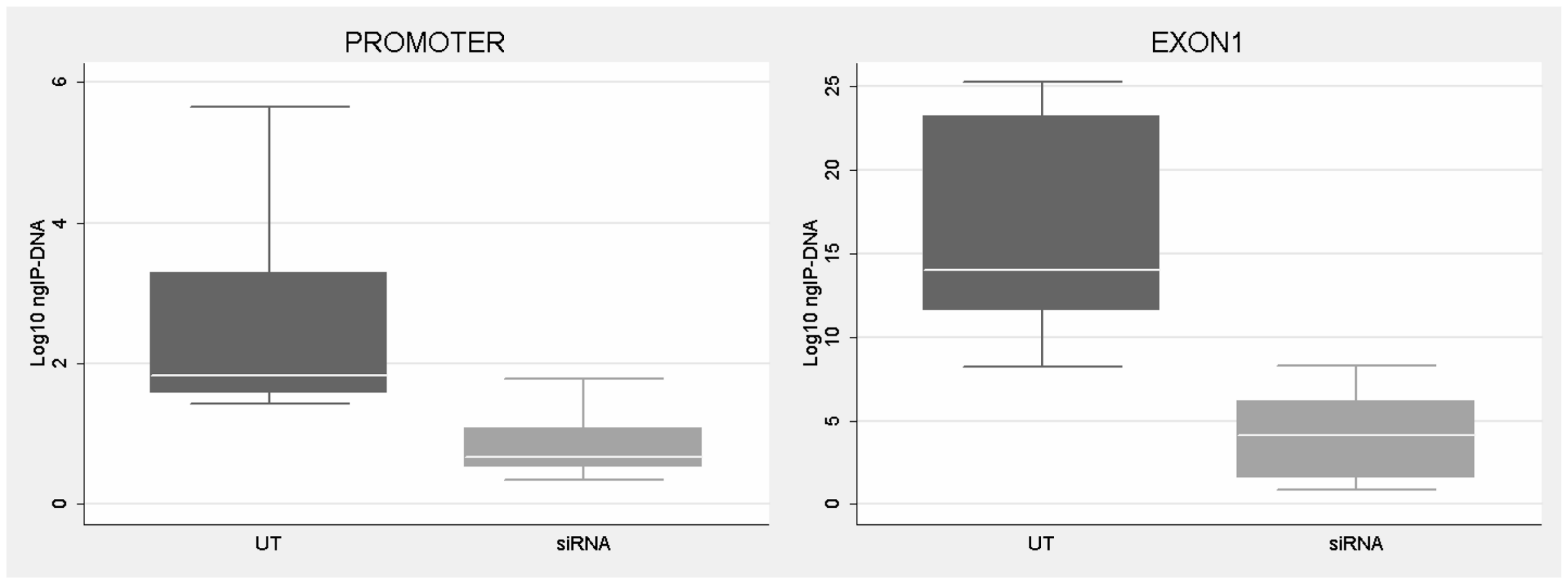
CTCF binding analysis on *FMR1* gene after *CTCF* knock-down. ChIP assay demonstrates the decrease of CTCF binding on *FMR1* promoter and exon 1 in WT fibroblasts after *CTCF* knock-down and *FMR1* reduction. Box-plots indicate the mean of at least three independent experiments, the corresponding standard error and standard deviation (thin lines). For both regions analyzed the level of CTCF binding in untreated WT (UT) is significantly higher respect to cells treated with siRNA against *CTCF* (siRNA) (p = 0.0003 for promoter region; p = 0.0001 for exon1). Note that the amount of IP-DNA (ng) is indicated on a logarithmic scale. ChIP experiments included negative controls performed by IgG immunoprecipitation and no template control (not shown).

We transfected synthetic siRNAs specific for *CTCF* transcript into WT and UFM fibroblasts to reduce *CTCF* mRNA and to verify the effect of this reduction on *FMR1* transcription. In each knock-down experiment *CTCF* mRNA depletion was confirmed by quantitative RT-PCR, in comparison with *GAPDH* mRNA levels, used as control (data not shown). The CTCF reduction was also confirmed on protein levels both in WT and UFM cells (**Figure 5A**). The residual *CTCF* transcription was around 15–20% in both fibroblast lines (**Figure 5B**). On the other hand, the effect on *FMR1* transcription was variable. In about two thirds of all knock-down experiments performed on both cell lines, no modification in *FMR1* transcription was observed, while in the remaining third we observed a near 50% reduction of *FMR1* transcription, as exemplified in **Figure 5B**. Interestingly, the *FMR1* mRNA decrease was accompanied by a similar reduction of the *FMR1-AS1* transcription in both cell lines (**Figure 5B**). We also found that *CTCF* knock-down coupled with *FMR1* reduction resulted in lower levels of *CTCF* bound to the *FMR1* sites in the promoter and exon 1 of WT cells (**Figure 6**). In those *CTCF* knock-down experiments in which *FMR1*-mRNA remained unmodified, ChIP assay demonstrated no variation in CTCF binding at the promoter and exon 1 in WT as well as in UFM cells (**Table 1**).

**Table 1:** ChIP results after *CTCF* knock-down without *FMR1* transcript reduction.

	WT		UFM	
	UT	siRNA	UT	siRNA
**Promoter**				
CTCF	0.81±0.6*	0.8±0.51*	0.22±0.06*	0.26±0.02*
H3-K4 methylation	1.82±0.69*	1.62±0.72*	1.36±0.13*	1.13±0.1*
H3-K9 methylation	1.27±0.09*	1.93±0.89*	1.42±0.13*	1.35±0.02*
**Exon1**				
CTCF	1.67±1.12	1.78±0.39	0.39±0.03*	0.21±0.08*
H3-K4 methylation	2.51±1.24	1.79±0.82	2.18±0.17*	1.6±0.15*
H3-K9 methylation	1.37±0.31*	1.57±1.3*	1.92±0.88*	1.23±0.28*

CTCF binding levels and H3-K4/H3-K9 methylation in WT and UFM fibroblasts before (UT) and after *CTCF* depletion (siRNA) not followed by *FMR1* transcript reduction for the promoter and exon 1 regions, respectively. All values correspond to the mean amount of IP-DNA (ng)±standard deviation.

* indicated statistically significant values (p<0.05).

**Figure 7:**
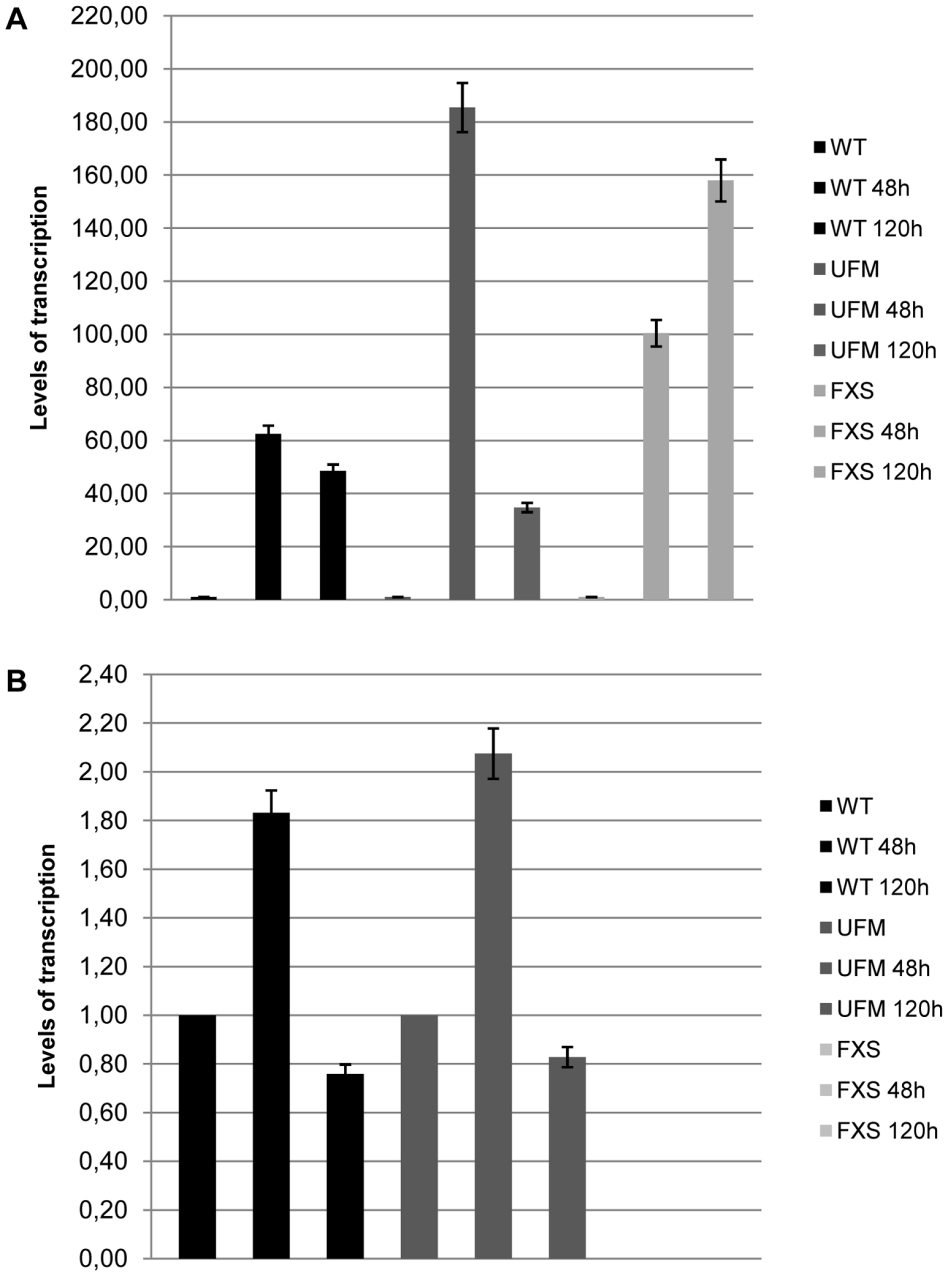
*CTCF* overexpression in WT, UFM and FXS fibroblasts. *CTCF* overexpression in WT, UFM and FXS cell lines after transfection with a commercial vector containing the open reading frame of variant 1 of *CTCF*. Quantitative RT-PCR showed a strong increase of *CTCF* mRNA after 48 and 120 hours from transfection (**A**), while levels of *FMR1* transcription remained substantially unchanged (**B**). The levels of *CTCF* transcription in the untreated cells were arbitrarily set at 1 as well as those of *FMR1* transcript in WT and UFM fibroblasts, while those of *FMR1*-mRNA in FXS cells were set at 0. Bars indicate standard deviation.

The next step was to establish whether overexpression of *CTCF* transcript could affect the transcription of *FMR1*. This was accomplished by transfecting a plasmid containing the variant 1 of human *CTCF* open reading frame into WT, UFM and FXS fibroblasts. The levels of overexpression ranged from 40 to 180 folds compared to the untreated controls, as confirmed by qRT-PCR (**Figure 7A**). Even in presence of the highest *CTCF* overexpression, the level of *FMR1* transcript remained substantially unmodified in all cell lines analyzed (**Figure 7B**).

### CTCF contributes to maintain the euchromatic status of the *FMR1* locus

**Figure 8:**
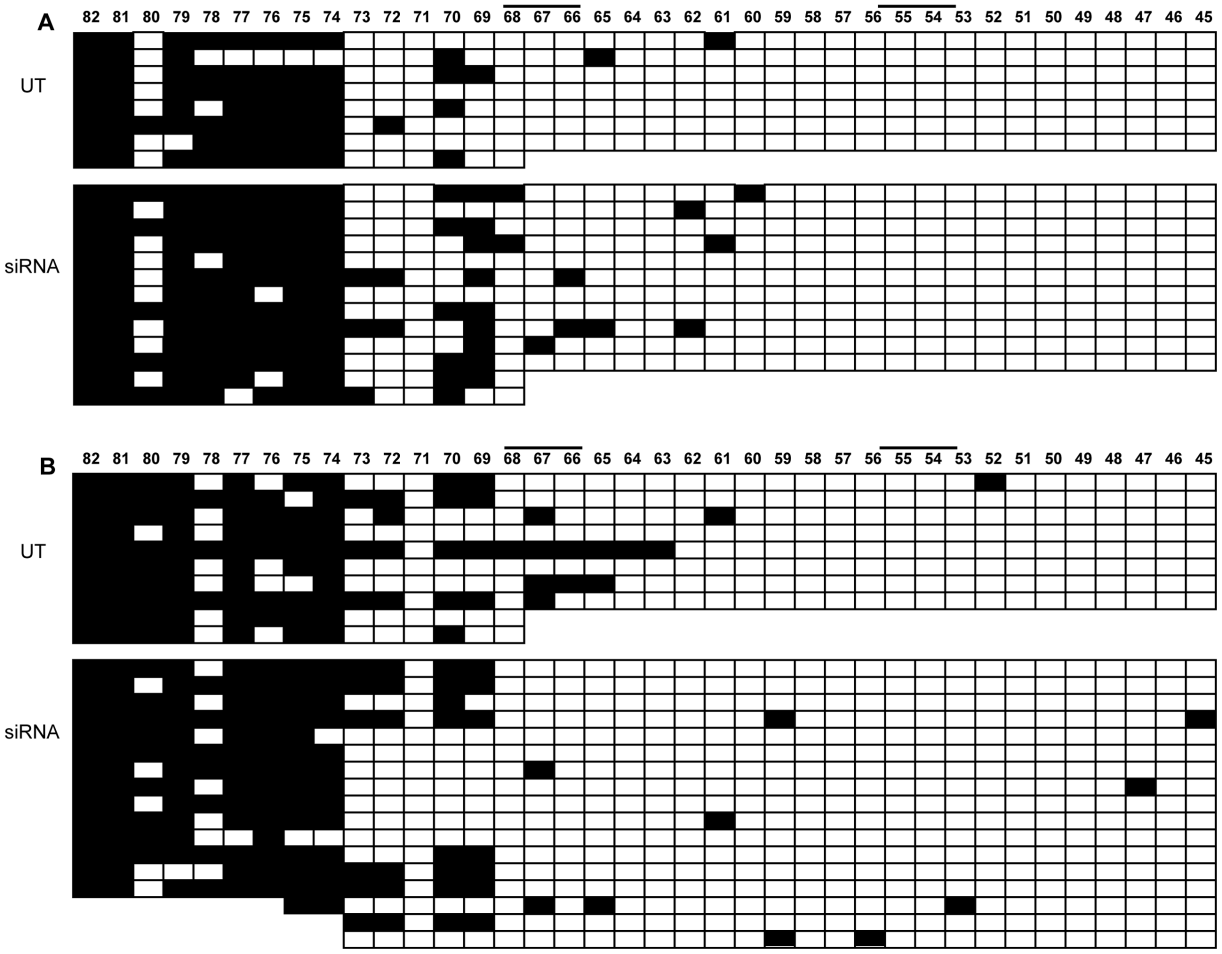
Methylation analysis of *FMR1* locus after *CTCF* knock-down. Bisulfite sequencing of 82 CpGs within the 5′-UTR of *FMR1* gene after *CTCF* knock-down in WT (**A**) and UFM (**B**) fibroblasts. Every line corresponds to bisulfite sequencing of an individual cell. Black and white squares correspond to methylated and unmethylated CpG sites, respectively. In this experiment the *FMR1* transcriptional reduction was around 30% with a residual 20% of *CTCF* transcript. In spite of *FMR1* transcriptional reduction (indicated as siRNA), there was no methylation spreading towards active *FMR1* promoter, that remained unmethylated as in an untreated control (UT). Note that CpG pairs between 45 and 54 are within the promoter region. Black bars indicate CTCF binding sites in the MB and in the promoter region.

To understand the molecular events underlying the variable results of *CTCF* knock-down experiments, we investigated the DNA methylation status and the chromatin organization of the *FMR1* locus after *CTCF* depletion coupled with *FMR1* reduction in WT and in UFM fibroblasts. Surprisingly, when we analyzed the methylation of promoter CpGs by bisulfite sequencing, all 52 CpGs were found unmethylated, as in the untreated controls. We extended our observation to the upstream region, observing that the methylation boundary persisted after *CTCF* depletion and *FMR1* transcript reduction (**Figure 8A and B**). Therefore, *CTCF* knock-down did not induce the spreading of methylation from the boundary to the *FMR1* promoter region, even in presence of a CGG expansion (**Figure 8B**).

**Figure 9:**
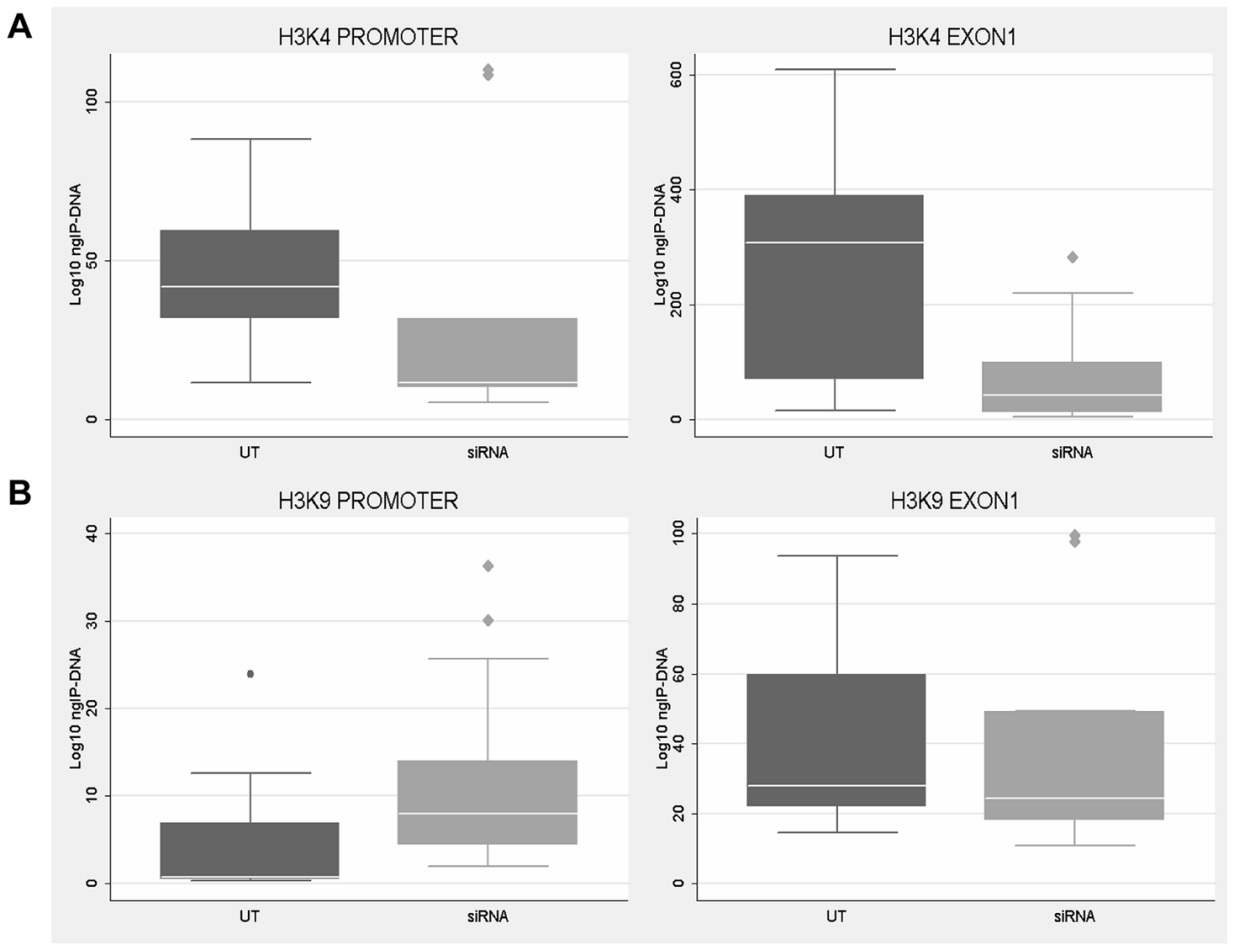
Histone H3 methylation analysis after *CTCF* knock-down. ChIP analysis of H3-K4 (**A**) and H3-K9 (**B**) methylation in WT fibroblasts after *CTCF* knock-down with *FMR1* reduction. Each box-plot depicts the amount (ng) of IP-DNA in promoter and exon 1 regions in control (UT) and siRNA transfected (siRNA) cells. The levels of H3-K4 methylation were significantly reduced in both regions (p<0.05), while those of H3-K9 were increased particularly in the promoter. Box-plots indicate the mean of at least three independent experiments, the corresponding standard error and standard deviation (thin lines). ChIP experiments included negative controls performed by IgG immunoprecipitation and no template control (not shown).

On the other hand, *FMR1* transcriptional reduction was found to correlate with histone epigenetic changes. In fact, in those experiments in which *CTCF* knock-down did not correlate with *FMR1* reduction, no variation of epigenetic marks (i.e. methylation of H3-K4 and H3-K9) was observed in the promoter and exon 1 of WT fibroblasts (**Table 1**). Instead, in those experiments in which *CTCF* knock-down correlated with *FMR1* transcript reduction, we observed a decreased methylation of H3-K4 in both regions analyzed (promoter and exon 1) and increased methylation of H3-K9 in the promoter region, compared to the untreated WT cells (**Figure 9**). These changes are representative of a more heterochromatic configuration of the locus, correlating with the reduction of *FMR1* transcription.

### Computational prediction of chromatin loops inside the *FMR1* locus

Our data support a mechanism of transcriptional co-regulation between *FMR1* sense and antisense, supporting a different role for CTCF protein rather than that of insulator. Based on the variability of *FMR1* transcription after CTCF knock-down, we shifted our focus on the role of this protein as chromatin organizer particularly in the loops formation. In order to explore the possibility that CTCF bound to its sites near the *FMR1* gene transcription start site (TSS) shapes regulatory chromatin loops, we performed a statistical and computational analysis of DNA structural properties of known regulatory loops determined by 5C experiments [35], compared to those of control genomic regions, and trained a machine learning algorithm to discriminate between real and control DNA loops (**Text S1** and **Figure S2**).

All putative CTCF-mediated loops in the proximity of the *FMR1* gene TSS were tested *in silico*, pairing the CTCF binding sites illustrated in **Figure 2**. We simulated the CGG expansion by adding 200 CGG triplets to the 5′UTR of the *FMR1* gene. The results of this predictions are reported in **Table 2**. All loops involving the intron 2 binding site, in which a *FMR1-AS1* transcriptional start site was identified, were predicted with high confidence both in WT and in the expanded allele. The *in silico* analysis excluded loops formation between exon 1 and all the other CTCF binding sites.

**Table 2:** Results obtained from SVM prediction system.

Putative Loops	Prediction	Probability	Prediction	Probability
	WT allele	WT allele	Expanded allele	Expanded allele
MR – exon 1	L	0.53	L	0.68
MB – exon 1	NL	0.96	NL	1
prom – exon 1	NL	1	NL	1
**MR – intron 2**	**L**	**0.84**	**L**	**0.87**
**MB – intron 2**	**L**	**0.9**	**L**	**0.92**
**prom – intron 2**	**L**	**0.91**	**L**	**0.92**
exon 1 – intron 2	NL	0.52	NL	0.51

The first column lists the possible combination of CTCF binding sites, as already reported (Figure 2). Columns 2 and 3 report the prediction (L = predicted loop, NL = predicted non-loop) and the probability of WT allele for each putative loop, while columns 4 and 5 report results of expanded CGG allele (>200 repeats). Probability is an accuracy index of prediction, higher is its value more confident is the prediction. In bold are reported the more probable loops.

## Discussion

Emerging evidence underlines the dynamic status of the chromatin, previously thought to be static, showing that a given region may be condensed (heterochromatin) and decondensed (euchromatin), according to the cell needs for transcriptional activity of that region. The discovery of proteins capable of establishing physical, as well as functional connections among distant genomic regions, even among different chromosomes, adds complexity to an already intricate network of gene-gene interactions. CTCF can be considered a leading candidate mediating these complex interactions [14]. In fact, it plays different roles in a gene-specific and context-specific manner depending on the possibility of creating homodimers and heterodimers with other proteins, such as cohesin, RNA Polymerase II and Parp1 [36]–[38]. CTCF was the first protein to be identified with a role of insulator, involved in the maintenance of the methylation boundaries in mammals [21]. Recently a methylation boundary region, which seems to prevent methylation to spread downstream, was reported in WT cell lines approximately 1 kb upstream the *FMR1* gene promoter, but not in FXS cells [13]. Other regions with this function were described in the myotonic dystrophy gene *DMPK*, in the ICR (Imprinted Control Region) of *IGF2* and in the neighboring *BLU* and *RASSF1A* loci of the 3p21.3 gene cluster region [30], [24], [39]. Triplet repeat expansion disorders often undergo transcriptional regulation by the CTCF protein, suggesting a role of CTCF also in *FMR1* gene transcriptional regulation.

Binding sites for CTCF in the *FMR1* locus were already identified [33], and now confirmed by our study, particularly in the promoter, exon 1 and intron 2, in which is located one of the transcriptional start site of the *FMR1-AS1*. We firstly showed that these three sites are bound to CTCF in UFM cells, both lymphoblasts and fibroblasts, and the binding level is quite similar to WT cells. These latter cell lines showed differences in CTCF binding in the two cell types analyzed (lymphoblasts and fibroblasts) and these variations should be related to differences between primary fibroblasts and Epstein-Barr-transformed and clonal lymphoblasts, as previously observed for other chromatin marks [4], [8], [27].

A CTCF binding site located in the *FMR1* methylation boundary was already described [33]. We now demonstrate for the first time the existence of the methylation boundary in UFM cells, supporting the hypothesis of a regulatory role played by the boundary region in preventing gene silencing. Interestingly, the CTCF binding site located in this border region, between CpG pairs 66 and 69 in WT cells, was also observed in UFM cell lines, but not in FXS cells, as expected given the CpGs methylation status of the latter.

We then tried to restore CTCF binding to the *FMR1* gene in FXS cell lines by inducing DNA demethylation with 5-azadC. DNA demethylation resulted in *FMR1* transcription reactivation as expected, while CTCF binding to its specific sites on promoter, exon 1 and boundary region was not restored. This result might be explained by failure of drug-induced DNA demethylation to reverse all modifications that occur during gene silencing. As observed on *p16* and *MLH1* gene, 5-azadC treatment did not completely restore normal histone code and post-translational modifications of DNA binding proteins to reestablish long-term expression [40], [41]. We previously observed that transcriptionally reactivated FXS cell lines restored epigenetic changes consistent with an euchromatic status, without fully reaching the euchromatic configuration typical of normal control cell lines [4]. We also demonstrated that 5-azadC-induced demethylation is partial and transient. After 4 weeks from 5-azadC withdrawal, the *FMR1* promoter resumed its methylated status [11]. Therefore it can be inferred that CTCF binding, even if it occurred after 5-azadC demethylation, would not by itself sufficient to maintain the unmethylated status of the *FMR1* gene.

These data seemed to suggest a functional role of the CTCF protein in regulating *FMR1* gene transcription. To investigate this potential role, we induced both silencing and overexpression of *CTCF* transcript. In those experiments in which siRNA-mediated *CTCF* knock-down did not correlate with *FMR1* transcript reduction, epigenetic marks (CTCF binding, H3-K4/H3-K9 methylation) were unmodified in promoter and exon 1 regions. On the other hand, the level of CTCF protein still bound to the gene was found reduced in *CTCF* knock-down experiments coupled with *FMR1* mRNA reduction. Moreover, *FMR1* decreased expression correlated with increased levels of heterochromatinic marks, such as H3-K4 demethylation and H3-K9 hypermethylation in the 5′ UTR of the gene. Interestingly, these epigenetic changes, known to favor heterochromatinic configuration, were not followed by the spreading of DNA methylation from the boundary region towards the *FMR1* promoter, not only in WT alleles, but also in UFM alleles, suggesting that a CGG expansion is not by itself sufficient to induce methylation, even in absence of CTCF. This latter result implies that CTCF does not work as an insulator at the *FMR1* locus. Therefore, other still unknown proteins must act as barrier elements in this specific region, as already hypothesized [13]. There are a number of boundaries that may function in a CTCF-independent manner through the binding of proteins known to act as transcriptional regulators, such as USF1 [42], YY1 and EVI1, or through non-coding RNAs [43]. Particularly, USF1 is one of the major transcription factors that bind the *FMR1* promoter region. Its binding is partially inhibited by DNA methylation and it might be a hypothetical candidate as insulator for the *FMR1* gene [44].

Interesting results came from the *FMR1* antisense transcript characterization, particularly in UFM cell lines, both before and after *CTCF* transcriptional silencing. The *FMR1* antisense RNA is transcribed starting from the second intron of the gene in WT and premutated alleles [33]. We detected, for the first time, *FMR1-AS1* RNA in UFM cell lines and also showed that the levels of this antisense transcript were higher in UFM cells, compared to normal controls, similar to what happens with the sense transcript [7]. The antisense transcript splices a 9.7 kb intron corresponding to the *FMR1* intron 1, that uses the complementary splice donor and acceptor to *FMR1*, representing a non-consensus CT to AC splice site. Moreover we observed in UFM cells the same splicing variant of the *FMR1-AS1* previously described as premutation-specific alternative splicing in intron 2 that also uses a non-consensus CT-AC splice site [33]. Furthermore, after *CTCF* depletion the reduction of *FMR1* mRNA was always coupled with the decrease of *FMR1-AS1* transcript. These data indicated a co-regulation of transcription and splicing mechanisms at the *FMR1* locus in transcriptional active alleles. On the other hand, *CTCF* knock-down did not have always the same effect: in only one third of all the experiments we observed a diminished transcription of both sense and antisense *FMR1*. These results suggested a partial and/or indirect role of CTCF in regulating *FMR1* expression and led us to hypothesize that the sites located within the *FMR1* locus may form chromatin loops mediated by CTCF homodimers capable of bringing in close proximity molecular machineries for transcription, splicing and epigenetic modifications. The formation of these loops would be partially affected by *CTCF* knockdown but not by *CTCF* overexpression, i.e. additional CTCF protein would not affect loop formation [45], [46]. Loss of CTCF-mediated chromosomal organization through disruption of this loop could exert a negative effect on *FMR1* transcription. On the other hand, it would seem that other factors, yet to be identified, could activate self-preserving mechanisms that maintain *FMR1* transcription unchanged despite the absence of the loop, as observed in a fraction of our experiments. Indeed, how chromatin configurations may influence gene expression still remains unclear. The “loop” hypothesis was supported by antisense transcription data, as well as by *CTCF* depletion/overexpression experiments. The presence of a CTCF binding site in *FMR1* intron 2, near one of the transcription starting sites of *FMR1-AS1*, previously observed by Ladd et al. [33], was confirmed in our cell lines by ChIP assays. Our hypothesis was that this CTCF site is involved in the chromatin looping together with one of the 5′-UTR sites within the active *FMR1* gene both in normal and in the expanded alleles, such as UFM. This loop may not form after 5-azadC-induced demethylation, which cannot reestablish native epigenetic modifications. In fact, as previously observed, 5-azadC effect is only transient [11]. The region surrounding the *FMR1* promoter (approximately 50 kb) was previously studied through 3C technique, which demonstrated reduced interaction frequencies [47]. This work did not take into account the behavior of the chromatin region surrounding the active *FMR1* gene with CGG expansion, such as in premutation and UFM cells. The 3C technique is only capable of detecting chromatin loop interactions greater than 10 kb and for this reason a chromatin loop formation in our region of interest cannot be excluded. We investigated the possibility of looping between CTCF sites using an *in silico* analysis of DNA structural characteristics of experimentally validated DNA regulatory loops. For this purpose, we elaborated a new predictor system that showed good performances in discriminating between real loops and control genomic regions. This predictor (SVM) confirmed that putative loops can form involving the CTCF binding site in intron 2, both in WT and in expanded alleles.

The bioinformatics approach takes into account parameters concerning the nucleotide sequence but not molecular and epigenetic characteristics, such as DNA methylation. *In silico* data should be interpreted considering the biological context in which the *FMR1* gene is located. Therefore, loop formation in FXS alleles was excluded by the existence of DNA methylation of the entire region upstream the *FMR1* promoter, that prevents CTCF from binding its sites. The formation of loops between intron 2 and MR sites could also be excluded because the MR site is located in a region that is extensive methylated in WT and in expanded alleles. Our *in silico* results affirmed that a chromatin loop mediated by CTCF homodimers can exist between intron 2 and the methylation boundary region or promoter in normal and UFM alleles. These bioinformatics data will deserve further experimental validations.

In conclusion our results delineate a role for CTCF as transcriptional regulator of *FMR1* expression through chromatin organization. CTCF was firstly described as the only known insulator [48], but we show that it does not act as an insulator on the methylation boundary upstream the *FMR1* gene. A role of CTCF in genome and locus organization acting to secure long-range intra- and inter-chromosomal interactions was abundantly described [22]. Our results define an indirect role for CTCF in modulating bidirectional transcription through *FMR1* locus chromatin organization and loop formation. Indeed, reduction of *FMR1* sense and antisense transcription after *CTCF* depletion underscores the importance of the CTCF-mediated loop complex. This study will be help in further clarifying the processes by which cell type specific patterns of gene expression can be established and maintained.

## Materials and Methods

### Cell lines and pharmacological treatments

Lymphoblastoid cell lines were established by Epstein–Barr virus transformation from peripheral blood lymphocytes of FXS, UFM and normal control (WT) males. The FXS cell lines employed in these experiments were E3 and S1, with 250 and 450 CGGs, respectively; the UFM cell line (MA) contains 265–430 CGGs [8]; two different WT cell lines obtained from normal control males. Lymphoblasts were grown in RPMI1640 medium (Sigma Aldrich) supplemented with 20% fetal bovine serum, 2.5% L-glutamine and 1% penicillin/streptomycin at 37°C with 5% CO_2_.

Primary fibroblast cultures were obtained from skin biopsies derived from the UFM individual (MA). We have also employed one FXS line (GM04026) and three WT lines (GM05381, GM03349 and GM07492), provided by the Coriell Institute (Camden, USA). Fibroblasts were grown in BIO-AMF2 complete medium (Biological Industries).

FXS lymphoblasts were treated with the demethylating agent 5-azadC (Sigma-Aldrich), as previously described [10]. Cells were seeded at 7×10^5^ cells/ml and 5-azadC was added daily at 1 µM (final concentration) for 7 days. At the end of the treatment, cells were harvested to measure viability with the propidium iodide method (Nucleocounter, Sartorius/Stedim) and to perform RNA and DNA extraction.

### Transfection experiments

Knock-down of *CTCF* transcripts was carried out in UFM and in all three WT fibroblast lines with synthetic siRNAs (Dharmacon, USA). Complete sequences of the siRNAs are listed in **Table S1**. Negative control to check the efficiency of *CTCF* depletion was performed using scramble siRNA (IDT). In accordance with the protocol of the manufacturer, 40 nM of siRNA were transfected by Lipofectamine RNAiMAX (Invitrogen, USA) and cultures were harvested after 72 hours.

The human open reading frame of *CTCF* was transfected into the cells through the expression plasmid pCMV6-Entry (C-terminal Myc- and DDK-tagged) (Origene). 100 ng of plasmid DNA was transfected in fibroblasts with Lipofectamine 2000 (Invitrogen, USA) and cells were collected after 48 h, according to manufacturer's instructions, and after 120 h to asses if a longer overexpression could affect *FMR1* transcription.

### Western blotting analysis

Proteins extracted from untreated and siRNA-treated WT and UFM fibroblasts were resuspended in Laemli buffer, boiled, separated on 8% polyacrylamide gel electrophoresis, transferred to Hybond-ECL membrane (GE Healthcare), immunostained and visualized after film exposure using the ECL Western Blotting Kit (GE Healthcare), according to the manufacturer. Primary antibodies were used at the following concentrations: 1∶1000 anti-CTCF rabbit policlonal antibody (Millipore) and 1∶10000 anti-GAPDH mouse antibody (Sigma-Aldrich).

### Methylation analysis

Genomic DNA was isolated from siRNA-treated and untreated fibroblasts both WT and UFM by DNeasy Blood & Tissue kit (Qiagen) The DNA concentration was checked both by absorbance measurements at 260 and 280 nm and on agarose gel. Bisulfite DNA transformation was performed as previously described [11]. Each transformed DNA was amplified in 7 independent PCR reactions, then pooled and recovered from the agarose gel with the StrataPrep DNA Gel extraction kit (Stratagene). The purified PCR products were cloned with the StrataClone PCR cloning kit (Stratagene), according to the manufacturer's instructions. After bacterial plating and overnight incubation at 37°C, white colonies were picked and plasmid DNA was extracted. After a pre-screening of the clones with PCR using specific plasmid primers (M13 forward and reverse), amplification products were sequenced in both directions with BigDye Terminator v3.1 Cycle Sequencing kit (Applied Biosystems) on a 3130 Genetic Analyzer (Applied Biosystems). The modified primers are those described by Naumann et al. [13].

### Quantitative RT-PCR analysis

Total RNA was extracted by TRIzol (Invitrogen, USA). RNA concentration and purity were checked on agarose gel and by UV spectrophotometer. RNA samples were treated with TURBO DNA-free DNase (Ambion) to remove contaminating DNA. Afterwards, 1 µg of total RNA was retro-transcribed into cDNA by MoMLV-RT (Invitrogen, USA) using random hexamers. For a relative quantification of each transcript, the following pre-developed TaqMan assays (Applied Biosystems) were used: *CTCF* (Hs00902008_m1), *GAPDH* (402869), *FMR1* (Hs00233632_m1). For *FMR1*-*AS1*, custom-made assay was designed (ASFMR1F 5′-CCTCTGCCAACTCAGTGCTATTAG-3′; ASFMR1R 5′-CATGACCTAGTCTGGGGTGGAG-3′; ASFMR1Probe 5′-(FAM)-TGGAATCATCTCCCC-(TAMRA)-3′(Applied Biosystems), according to Ladd et al. [33]. The real-time RT-PCR was performed on a ABI7900HT (Applied Biosystems). The cycle parameters were: 2 minutes at 50°C and 10 minutes at 95°C, followed by 40 cycles with 15 seconds at 95°C (denaturation) and 1 minute at 60°C (annealing/extension).

### Strand-specific RT-PCR

To analyze the *FMR1*-*AS1* transcript, cDNA was generated using specific primers, with a linker (LK) sequence: 5′-CGACTGGAGCACGAGGACACTGA-3′attached to the 5′ end. Primers were those employed by Ladd et al. [33]. cDNA was produced using Superscript III (Invitrogen), according to the manufacturer instruction's. PCR were performed using the LK primer (as forward) and antisense specific reverse primers. The amplicons were sequenced on an 3130 Genetic Analyzer (Applied Biosystems).

### Chromatin Immunoprecipitation (ChIP) and quantification of IP-DNA

ChIP assay was performed according to the manufacturer (Upstate Biotechnology, USA). After 10 minutes at 37°C with 1% formaldehyde, cells were seeded and washed with 1× PBS and Protease Inhibitor Cocktail (Sigma-Aldrich). To obtain 200–1000 bp DNA fragments, cell pellets were sonicated. Histone methylation analysis was performed using two different antibodies against dimethyl lysine 9 (H3-K9, 07–441, Upstate Biotechnology) and dimethyl lysine 4 (H3-K4, 07–030, Upstate Biotechnology) on histone 3. Binding of CTCF protein was assayed using the specific antibody (07-729, Millipore). In each ChIP assay antibody against rabbit IgG (1862244, Thermo Scientific) was employed and also no template control was included. Immunoprecipitated DNA (IP-DNA) was extracted by standard procedure (phenol/chloroform/isoamilic alcohol 25∶24∶1) and then quantified by real-time PCR (ABI7900HT, Applied Biosystems) using fluorescent probe and primers specific for both *FMR1* and *HPRT*.

Primers and probes employed for PCR analysis are listed in **Table S2**. Standard curves for the three *FMR1* and for the single *HPRT* amplicon were constructed with five different DNA dilutions of known concentration (X axis = log[X]) and the corresponding Ct values (Y axis). The unknown amount of methylated histone and CTCF-binding IP-DNA of *FMR1* and *HPRT* (X axis = log[X]) was calculated from Ct values, through the standard curve plot. Normalized *FMR1* levels were estimated dividing the amount of *FMR1* IP-DNA by the amount of *HPRT* IP-DNA.

### Statistical analysis

All variables were analyzed by means of descriptive statistics (mean, median, standard deviation and standard error of mean). Data were analyzed with non-parametric statistical Kruskal-Wallis test and with K sample test. The level of significance was set at p≤0.05. Data analysis was performed using STATA Intercooled v. 9.2 software (Stata Co.; College Station, Lakewag, TX, USA).

### Computational structural analysis and prediction of CTCF-mediated DNA loops

In order to analyze the structural characteristics of CTCF-mediated DNA loops, a bioinformatics approach was developed and is detailed in the **Text S1**. Briefly, a machine learning method was trained to recognize known chromatin loops from control genomic regions, and then used to test putative regulative loops in the proximity of *FMR1* transcription start site.

Supplementary Data are available online: Supplementary Figures S1, S2, Supplementary Tables S1, S2, Supplementary Text S1 and Supplementary References S1 [34], [35],[49]–[58].

## Supporting Information

Figure S1 Methylation boundary region analysis. Bisulfite sequencing of the methylation boundary region of *FMR1* gene in WT, UFM and FXS cell lines: lymphoblasts (**A**) and fibroblasts (**B**). Every line corresponds to bisulfite sequencing of an individual cell. Black and white squares correspond to methylated and unmethylated CpG sites, respectively. CpG pairs between 45 and 54 are within the promoter region, whereas CpGs between 55 and 82 are located upstream. UFM cells present a transitional region of methylation similar to WT cells; in FXS cells this methylation boundary is completely lost. Black bars indicate CTCF binding sites in the MB and in the promoter region.
(TIF)

Figure S2 Computational analysis for *FMR1* locus chromatin conformation. Parameter distribution between real DNA loops (in the POS dataset) and both the random genomic controls (NEG1) and the CTCF-related controls (NEG2). The POS loops appear more bendable than NEG1 controls, but less than the NEG2 ones. A similar behavior can be observed for the DNA cleavage intensity, while the POS loops seem to be more stable to thermal denaturation than both controls. POS loops appear to have a lower average curvature than random genomic regions, and curvature values for POS loops were strongly inversely correlated to their bendability index (Pearson's correlation coefficient −0.9). This observation is not surprising since curved DNA is often the result of the interaction with chromatin proteins, and the associated entropy reduction is less unfavorable for less flexible DNA.
(TIF)

References S1 List of references included in the Text S1.
(DOC)

Text S1 Methodological details and performance evaluation for chromatin loops inside the *FMR1* locus. We analyzed DNA structural properties of known CTCF-mediated regulatory loops determined by 5C experiments (POS dataset) [35], compared to those of control genomic regions (NEG1 and NEG2), and trained a machine learning algorithm to discriminate between real and control DNA loops. A Support Vector Machine (SVM) was employed to test putative CTCF-mediated loops in the proximity of the *FMR1* gene TSS, pairing the CTCF binding sites illustrated in **Figure 2**.
(DOC)

Table S1 List of siRNA against *CTCF* transcript with the corresponding sequences.
(DOC)

Table S2 Primers and probes used for qPCR after ChIP assays.
(DOC)
