# Supplementary figures and images for: Role of CTCF Protein in Regulating FMR1 Locus Transcription
Source: PLoS Genet. 2013 Jul 18;9(7):e1003601. doi: 10.1371/journal.pgen.1003601 (PMC3715420; doi:10.1371/journal.pgen.1003601)

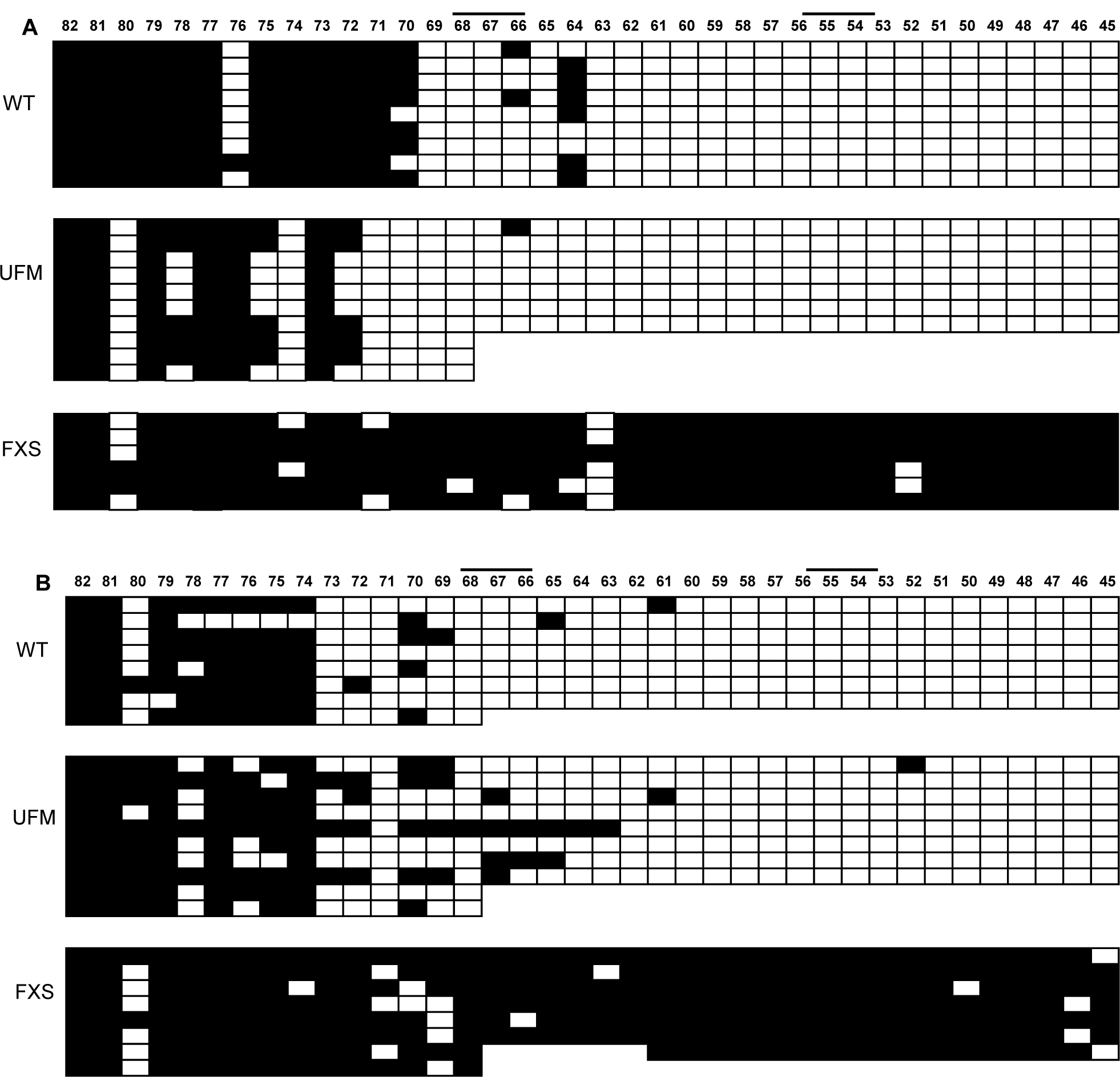

Supplement: Figure S1 — Methylation boundary region analysis. Bisulfite sequencing of the methylation boundary region of FMR1 gene in WT, UFM and FXS cell lines: lymphoblasts (A) and fibroblasts (B). Every line corresponds to bisulfite sequencing of an individual cell. Black and white squares correspond to methylated and unmethylated CpG sites, respectively. CpG pairs between 45 and 54 are within the promoter region, whereas CpGs between 55 and 82 are located upstream. UFM cells present a transitional region of methylation similar to WT cells; in FXS cells this methylation boundary is completely lost. Black bars indicate CTCF binding sites in the MB and in the promoter region. (TIF) [file pgen.1003601.s001.tif]

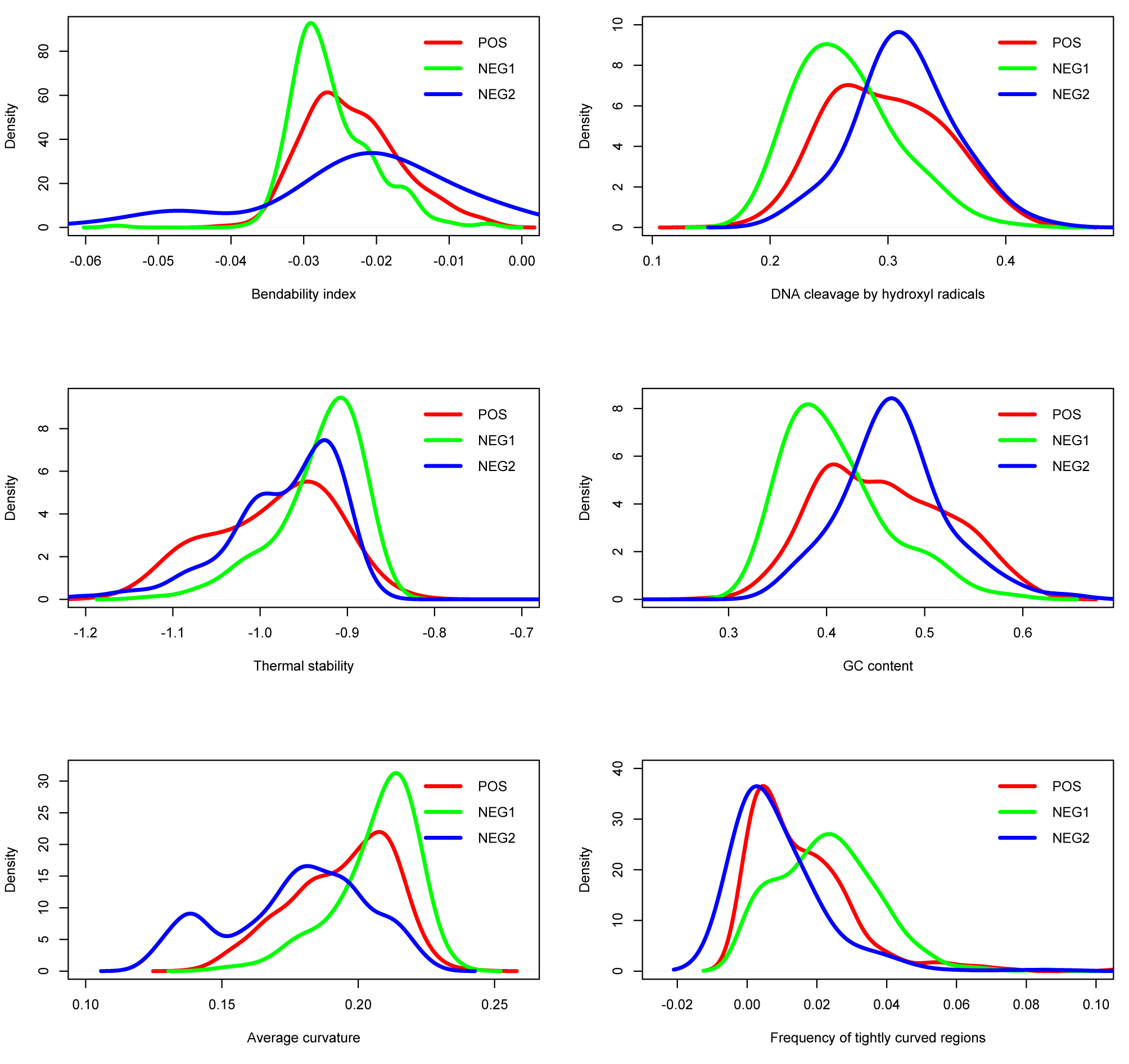

Supplement: Figure S2 — Computational analysis for FMR1 locus chromatin conformation. Parameter distribution between real DNA loops (in the POS dataset) and both the random genomic controls (NEG1) and the CTCF-related controls (NEG2). The POS loops appear more bendable than NEG1 controls, but less than the NEG2 ones. A similar behavior can be observed for the DNA cleavage intensity, while the POS loops seem to be more stable to thermal denaturation than both controls. POS loops appear to have a lower average curvature than random genomic regions, and curvature values for POS loops were strongly inversely correlated to their bendability index (Pearson's correlation coefficient −0.9). This observation is not surprising since curved DNA is often the result of the interaction with chromatin proteins, and the associated entropy reduction is less unfavorable for less flexible DNA. (TIF) [file pgen.1003601.s002.tif]

**
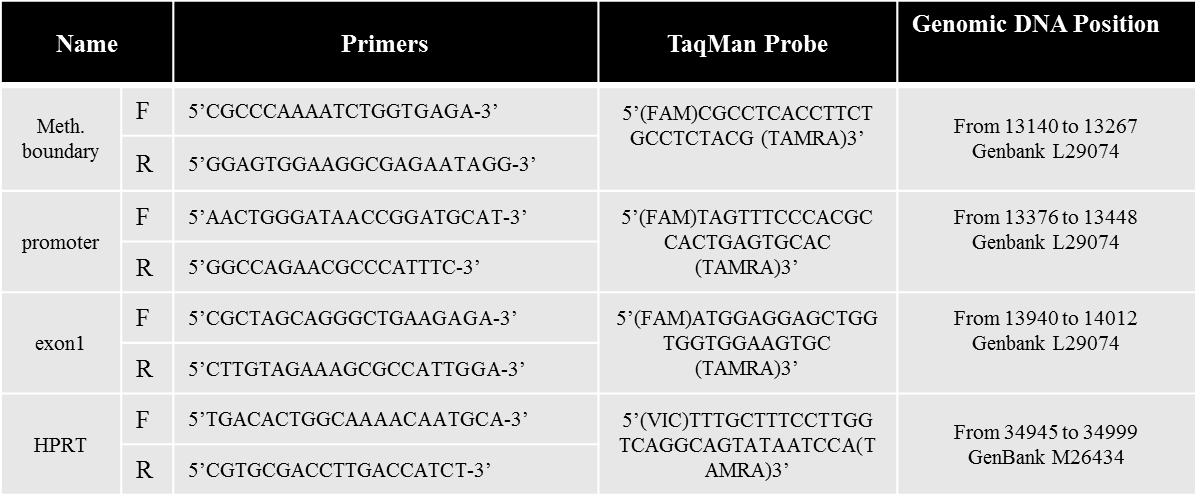
**

Supplement: Table S2 — Primers and probes used for qPCR after ChIP assays. (DOC) [file pgen.1003601.s006.doc]
