# Supplementary material for: Role of CTCF Protein in Regulating FMR1 Locus Transcription
Source: PLoS Genet. 2013 Jul 18;9(7):e1003601. doi: 10.1371/journal.pgen.1003601 (PMC3715420; doi:10.1371/journal.pgen.1003601)
Supplement: References S1 — List of references included in the Text S1. (DOC) [file pgen.1003601.s003.doc]

Supplementary references S1

1. Dreszer TR, Karolchik D, Zweig AS, Hinrichs AS, Raney BJ et al. (2012) The UCSC Genome Browser database: extensions and updates 2011. Nucleic Acids Res 40(Database issue): D918-23
2. ENCODE Project Consortium (2011) A user's guide to the encyclopedia of DNA elements (ENCODE). PLoS Biol 9: e1001046.
3. Flicek P, Amode MR, Barrell D, Beal K, Brent S et al. (2012) Ensembl 2012. Nucleic Acids Res 40 (Database issue): D84-90.
4. Li W, Godzik A (2006) Cd-hit: a fast program for clustering and comparing large sets of protein or nucleotide sequences. Bioinformatics 22: 1658-1659.
5. Brukner I, Sánchez R, Suck D, Pongor S (1995) Sequence-dependent bending propensity of DNA as revealed by DNase I: parameters for trinucleotides. EMBO J 14: 1812-1818.
6. Bishop E, Rohs R, Parker S, West S, Liu P et al. (2011) A map of minor groove shape and electrostatic potential from hydroxyl radical cleavage patterns of DNA. ACS Chem Biol 6: 1314–1320.
7. Shpigelman E, Trifonov E, Bolshoy A (1993) CURVATURE: software for the analysis of curved DNA. CABIOS 9: 435–444.
8. Trifonov EN, Ulanovsky LE (1987) Unusual DNA Structures. Wells RD. Harvey, S.C, Springer-Verlag, Berlin.
9. SantaLucia J Jr (1998) A unified view of polymer, dumbbell, and oligonucleotide DNA nearest-neighbor thermodynamics. Proc Natl Acad Sci USA 95: 1460-1465.
10. Chang CC, Lin CJ (2011) LIBSVM: a library for support vector machines. ACM Transactions on Intelligent Systems and Technology (TIST) 2: 1-27.
