# Supplementary material for: Role of CTCF Protein in Regulating FMR1 Locus Transcription
Source: PLoS Genet. 2013 Jul 18;9(7):e1003601. doi: 10.1371/journal.pgen.1003601 (PMC3715420; doi:10.1371/journal.pgen.1003601)
Supplement: Text S1 — Methodological details and performance evaluation for chromatin loops inside the FMR1 locus. We analyzed DNA structural properties of known CTCF-mediated regulatory loops determined by 5C experiments (POS dataset) [35], compared to those of control genomic regions (NEG1 and NEG2), and trained a machine learning algorithm to discriminate between real and control DNA loops. A Support Vector Machine (SVM) was employed to test putative CTCF-mediated loops in the proximity of the FMR1 gene TSS, pairing the CTCF binding sites illustrated in Figure 2 . (DOC) [file pgen.1003601.s004.doc]

**Methodological details and performance evaluation for chromatin loops inside the *FMR1* locus**.

To analyze the structural characteristics of CTCF-mediated DNA loops in the proximity of the *FMR1* gene transcription start site (TSS), data from Chromatin Conformation Capture Carbon Copy (5C) [35] were downloaded from the UCSC Genome Browser [49]. CTCF binding sites, determined by ChIP-seq, were downloaded from UCSC and from the CTCFBSDB [34], excluding predicted binding sites. Chromatin interaction data were extracted from 5C experiments, depicting the formation of loops between TSSs and distal regulatory elements in the context of the genomic regions investigated in the ENCODE project [50]. 5C data consisted of 4489 loops detected with significant frequency in the analyzed samples. In a large fraction (74%) of such loops, a ChIP-seq determined CTCF binding site is found nearby both ends of the loop. The loop length distribution shows a rather large range (mean length 325128 bp, standard deviation 293416 bp). A subset of 308 5C-determined loops shorter than 50 Kb and having a CTCF binding sites in close proximity (at most 2500 bp) from both ends of the loop was selected for the structural analysis (the POS dataset). We additionally generated two control datasets of genomic regions not likely to form a loop, NEG1 and NEG2. The NEG1 control dataset was created by selecting the same number (308) of genomic regions starting from a random genomic coordinate in the GRCh37/hg19 human genome assembly and ending at a distance sampled by a normal distribution having mean and standard deviation equal to that of the POS dataset (29558 and 13998 bp, respectively). The NEG2 control dataset was created by sampling 308 distinct CTCF binding site pairs (not involved in the formation of a 5C-detected loop) at genomic distance at most one standard deviation from the mean length of the POS dataset, and requiring that at least one of the two CTCF binding sites is at most at 2 Kb distance from the TSS of a known human transcript, as reported in the Ensembl annotations (release 65) [51]. We additionally verified that no pair of genomic regions in either the NEG1 or NEG2 datasets shares more than 80% sequence identity using Cd-hit [52]. A number of structural and sequence parameters were computed for the POS, NEG1 and NEG2 datasets, and for the putative CTCF-mediated loops nearby the *FMR1* TSS: (i) Bendability profiles were calculated using bending propensity parameters for DNA trinucleotides [53] and smoothed using a 50 bp window; (ii) Hydroxyl radicals cleavage intensity were estimated using the ORChID2 algorithm [54] that predicts cleavage intensity of each loop in the analyzed datasets, computed from parameters for a set of tetranucleotide patterns from both strands of the DNA duplex; cleavage intensity for each nucleotide in the DNA sequence was smoothed using a 50 bp window; (iii) The CURVATURE algorithm [55] was used to compute the average curvature of 125 bp overlapping windows in the input DNA sequence; we also computed the frequency of local regions having curvature at least 0.34 cu (curvature units) [56], corresponding to the curvature of a segment of 125 bp of length with a shape close to a half-circle; (iv) DNA thermal stability was calculated using the unified nearest-neighbors parameters [57], in windows of 15 bp. For each structural parameter, we compared the distribution of its values computed for each sequence in the POS against that for either the NEG1 or NEG2 datasets, using the Student’s t-test or the Mann–Whitney non-parametric test (when the distributions are rejected by the Anderson-Darling test for normality). All statistical tests were performed using the R statistical environment implementations. Statistical comparison of the distribution of bendability index, DNA cleavage intensity, curvature and stability are significantly different (p-value < 0.05) when comparing POS vs. NEG1 and POS vs. NEG2, suggesting that these parameters could capture DNA structural characteristics that are necessary for the formation of a regulatory loop (**Figure S2**).

A Support Vector Machine (SVM) was used to discriminate between CTCF-mediated loops in the POS dataset and in control genomic regions in NEG1 and NEG2. SVM training, testing and predictions were performed using the LIBSVM library [58]. The radial basis function kernel parameters C and gamma were optimized using a grid search. A ten-fold cross-validation was used for training and testing. Each loop in the POS or either NEG1 or NEG2 datasets was encoded by a 70 attributes vector, one attribute each for bendability, global and local curvature, likelihood of DNA cleavage by hydroxyl radicals, thermal stability, GC content, and 64 for the relative frequency of all possible trinucleotides. The same encoding was used for all putative *FMR1* CTCF-mediated loops. Confusion matrices were generated from the POS vs NEG1 and POS vs NEG2 cross-validations. Prediction accuracy was estimated by the commonly used measures accuracy, sensitivity, sensibility, Matthews Correlation coefficient, and AUC (area under the ROC curve). After radial basis function kernel parameters optimization and ten-fold cross-validation, the SVM is able to discriminate between the POS and NEG1 datasets with accuracy 0.72, sensitivity 0.7, specificity 0.74 and Matthews’s correlation coefficient (MCC) 0.44, while the area under the receiver operating characteristic (ROC) curve (AUC) is 0.79. Surprisingly, the performance is even better when comparing the POS with the NEG2 dataset (accuracy 0.77, sensitivity 0.93, specificity 0.62, MCC 0.58, AUC 0.84).
