# Supplementary material for: Role of CTCF Protein in Regulating FMR1 Locus Transcription
Source: PLoS Genet. 2013 Jul 18;9(7):e1003601. doi: 10.1371/journal.pgen.1003601 (PMC3715420; doi:10.1371/journal.pgen.1003601)
Supplement: Table S1 — List of siRNA against CTCF transcript with the corresponding sequences. (DOC) [file pgen.1003601.s005.doc]

| **si-RNA** | **Sequence** |
| --- | --- |
| **1F**  **1R** | **5’-gaugaagacugaaguaauguu-3’**  **5’-cauuacuucagucuucaucuu-3’** |
| **2F**  **2R** | **5’-ggagaaacgaagaagaguauu-3’**  **5’-uacucuucuucguuucuccuu-3’** |
| **3F**  **3R** | **5’-gaagaugccugccacuuacuu-3’**  **5’-guaaguggcaggcaucuucuu-3’** |
| **4F**  **4R** | **5’-gaacagcccauaaacauaguu-3’**  **5’-cuauguuuaugggcuguucuu-3’** |
